# Hypoxia-induced autophagy mediates cisplatin resistance in lung cancer cells

**DOI:** 10.1038/srep12291

**Published:** 2015-07-23

**Authors:** Hui-Mei Wu, Zi-Feng Jiang, Pei-Shan Ding, Li-Jie Shao, Rong-Yu Liu

**Affiliations:** 1 Department of Pulmonary, Anhui Geriatric Institute, the First Affiliated Hospital of Anhui Medical University, Jixi Road 218, Hefei 230022, China

## Abstract

Hypoxia which commonly exists in solid tumors, leads to cancer cells chemoresistance via provoking adaptive responses including autophagy. Therefore, we sought to evaluate the role of autophagy and hypoxia as well as the underlying mechanism in the cisplatin resistance of lung cancer cells. Our study demonstrated that hypoxia significantly protected A549 and SPC-A1 cells from cisplatin-induced cell death in a Hif-1α- and Hif-2α- dependent manner. Moreover, compared with normoxia, cisplatin-induced apoptosis under hypoxia was markedly reduced. However, when autophagy was inhibited by 3-MA or siRNA targeted ATG5, this reduction was effectively attenuated, which means autophagy mediates cisplatin resisitance under hypoxia. In parallel, we showed that hypoxia robustly augmented cisplatin-induced autophagy activation, accompanying by suppressing cisplatin-induced BNIP3 death pathways, which was due to the more efficient autophagic process under hypoxia. Consequently, we proposed that autophagy was a protective mechanism after cisplatin incubation under both normoxia and hypoxia. However, under normoxia, autophagy activation ‘was unable to counteract the stress induced by cisplatin, therefore resulting in cell death, whereas under hypoxia, autophagy induction was augmented that solved the cisplatin-induced stress, allowing the cells to survival. In conclusion, augmented induction of autophagy by hypoxia decreased lung cancer cells susceptibility to cisplatin-induced apoptosis.

Lung cancer is the leading cause of cancer-related death worldwide^1^. The main principle of lung cancer therapy is to induce cell death or to inhibit cell survival^2^. The standard therapy of intermediate and advanced lung cancer is based on the combination of cisplatin and other chemotherapy agents^3^,^4^. Cisplatin is a potent DNA-damaging anticancer agent, and its major pharmacological effect is to induce cancer cell apoptosis^5^,^6^,^7^. However, the prognosis is considered poor particularly for patients with advanced stage due to the chemoresistance, in which hypoxic microenviroment potentially plays a critical role^8^. Hypoxia, which commonly presents in solid tumors, is due to the proliferation of tumor cells outpaces the blood vessel formation in the tumor mass^9^. To further explore the mechanism how hypoxic context influences the cisplatin intervention may improve the prospect of effective anti-cancer therapy.

Rationally, in response to stresses such as hypoxia or cisplatin, cells are potentially injured^10^,^11^. However, cells exhibit an elaborate regulated process termed “autophagy” to cope with these stresses^12^,^13^. Autophagy allows energy supply during starvation, thus has been defined as a protective mechanism^14^. Racent studies revealed that hypoxia is able to modulate autophagy, thereby increasing cell survival and chemoresistance^15^,^16^,^17^,^18^. Effective autophagy may inhibit cell death by exerting an influence on apoptosis^10^,^19^,^20^,^21^. Contrarily, inhibition of autophagy may promote cell death by potentiating cisplatin-induced apoptosis^18^,^22^.

It has been reported that the molecular pathways regulating autophagy and apoptosis are interconnected^23^. Moreover, autophagic and apoptotic pathways share several key molecular regulators, the modulation of one mechanism influences the execution of the other and vice-versa. Therefore, how hypoxia and autophagy work together to modulate cancer cells response to chemotherapy-induced apoptosis is complex. This study is aimed to reveal how hypoxia and autophagy work together to mediate cisplatin resistance in lung cancer cells.

## Results

### Hypoxia enhanced the cisplatin resistance of lung cancer cells

**Figure 1:**
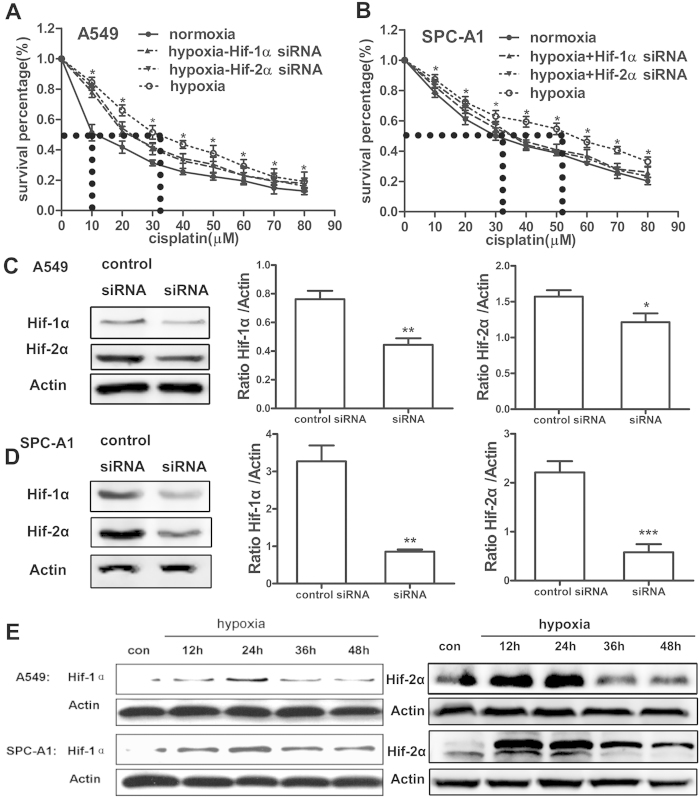
Hypoxia reduced chemosensitivity of lung cancer cells to cisplatin in a Hif-1α and Hif-2α dependent manner. Lung cancer cell lines A549 (**A**) and SPC-A1 (**B**) were incubated under normoxic (21%O_2_) and hypoxic (1%O_2_) condition with various concentrations of cisplatin for 24 h in the presence or absence of Hif-1α or Hif-2α siRNA. At the end of the treatment, cell viability was assessed by MTT. (**C**) The knockdown efficiency of Hif-1α or Hif-2α siRNA in the A549 cell. The IC50 was indicated by the dashed line. (**D**) The knockdown efficiency of Hif-1α or Hif-2α siRNA in the SPC-A1 cell. (**E**) Cells incubated with hypoxia at difference time point were harvested for protein extraction and then subjected to western blot using Hif-1α and Hif-2α antibody. The results were shown as means ± SD of four independent experiments. *p < 0.05, **p < 0.01, ***p < 0.001. Blot images were cropped for comparison.

For the lung is the first-line to contact with the atmosphere, in which the oxygen content is 21%, so 21% O2 is commonly used as normoxic condition^23^,^24^,^25^,^26^, while 1% O2 or 0.5% O2^26^ is usually applied as hypoxic condition. In the present study, to study the role of hypoxia on cisplatin resistance of lung cancer cells, A549 and SPC-A1 cells were maintained in complete medium at 21% (normoxia) or 1% O_2_ (hypoxia) for 24 h in the presence or absence of cisplatin. Cell viability assay by MTT showed that hypoxia significantly increased cell viability upon treatment of cisplatin, as compared with that in cells under normoxic condition (Fig. 1A,B), while this increase was markedly attenuated by pre-transfected cells with Hif-1α or Hif-2α siRNA (Fig. 1A,B). These results were also supported by PI staining (Supplementary Fig. 1), suggesting that hypoxia, to some extent, protects cells from cisplatin-induced cell death^27^,^28^. Additionally, The IC50 of cisplatin was determined in order to obtain an effective concentration for the further study. The IC50 of cisplatin for A549 and SPC-A1 cells under hypoxia was 3.38-fold and 1.57-fold higher as compared to that under normoxia respectively as showing by the dashed line in Fig. 1A,B. The cisplatin concentration closest to the IC50 was utilized for further analysis: 10 μM for A549 cells and 30 μM for SPC-A1 cells.

Figure 1C,D showed that transfection of A549 and SPC-A1 cells with 50pmol Hif-1α or Hif-2α siRNA significantly reduced the expression of Hif-1α or Hif-2α. Furthermore, the expression of Hif-1α and Hif-2α was analyzed by western blot to test the effect of the modular incubation chamber on the concentration of oxygen. As shown in Fig. 1E and supplementary Fig. 2, the expression of Hif-1α and Hif-2α was increased along with the time that cells exposed to hypoxic condition within 24 h.

### Hypoxia blocked cisplatin-induced subcellular redistribution of pro-apoptotic proteins

**Figure 2:**
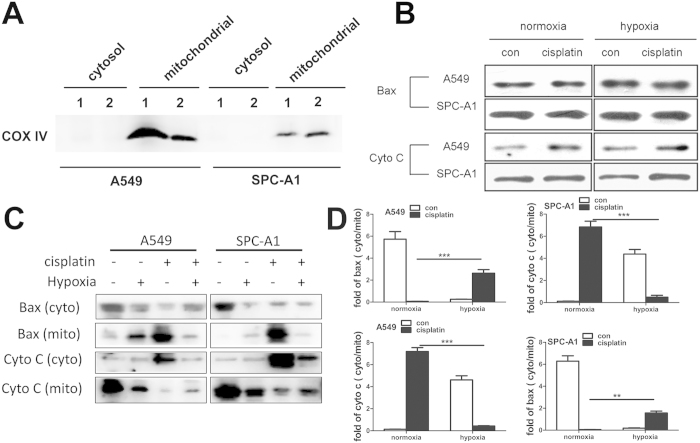
Hypoxia blocked cisplatin-induced subcellular redistribution of pro-apoptotic proteins in lung cancer cells. A549 and SPC-A1 cells were treated as indicated and then cells were fractionated into cytosolic fraction and the mitochondrial fraction. (**A**) COX IV protein expression in the cytosolic fraction and mitochondrial fraction (lane 1: normoxia, lane 2: hypoxia). (**B**) The total protein expressions of Bax and Cyto C. (**C**) The protein expression of Bax and Cyto C in cytosol and mitochondria. (**D**) The ratio of the expression of Bax or Cyto C in the cytoplasm to the expression of Bax or Cyto C in the mitochondira. The results were shown as means ± SD of three independent experiments. **p < 0.01, ***p < 0.001. Blot images were cropped for comparison.

**Figure 3:**
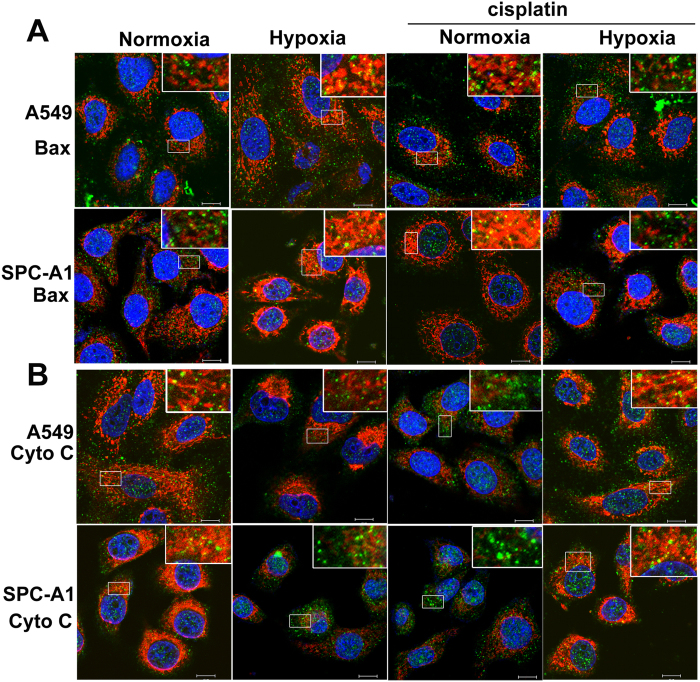
Hypoxia suppressed cisplatin-induced Bax translocation and Cyto C release. A549 and SPC-A1 cells were treated as mentioned above, then cells were immunostained by Bax or Cyto C antibody following MitoTracker staining. (**A**) Bax cellular localization. (**B**) Cyto C cellular localization. Representative images were from three independent experiments. Green: Bax or Cyto C; Red: mitochondria; Blue: nucleus; the scale bar: 10 μm.

Cisplatin-induced cell death in lung cancer cells potentially involves multiple signaling pathways. Among these pathways, mitochondrial apoptosis events (Bax translocation and Cyto C release) play a critical role. Here, we investigated the effect of hypoxia on these two cisplatin-induced mitochondrial apoptosis events. Cytosolic and mitochondrial proteins were separated and analyzed by western blot. The effect of separation was measured by detection of the expression of COX IV (Fig. 2A). The total protein levels of Cyto C and Bax showed no significant differences in normoxic group and hypoxic group with or without cisplatin treatment (Fig. 2B). However, Bax was detected primarily in the cytoplasm in the control group, but translocated from cytoplasm to mitochondria after 12 h hypoxia or cisplatin incubation, of interest was that the cisplatin-induced Bax translocation was significantly decreased in the presence of hypoxia in both A549 and SPC-A1 cells (Fig. 2C,D). On the other hand, hypoxia or cisplatin significantly induced Cyto C released from mitochondria into the cytoplasm, but the cisplatin-induced Cyto C release was suppressed by simultaneous treatment of hypoxia (Fig. 2C,D). Identical results were obtained by Bax and Cyto C immunocytochemistry with Confocal Laser Scanning Microscopy (CLSM) (Fig. 3A,B and supplementary Fig. 3). These results showed that hypoxia blocked cisplatin-induced subcellular redistribution of pro-apoptotic proteins, suggesting that hypoxia induces cisplatin resistance mainly by reducing lung cancer cells apoptotic potential.

### Inhibition of autophagy restores lung cancer cells sensitivity to cisplatin under hypoxia

Data above proved that hypoxia protected A549 and SPC-A1 cells from cisplatin, and this may be due to the decreased apoptotic potential. But how does it work? It has recently been reported that under adverse conditions such as hypoxia, cancer cells make themselves be adaptive by activating autophagy, thus we hypothesized that autophagy may attribute to hypoxia-induced cisplatin resistance in lung cancer cells. In order to test this hypothesis, we inhibited autophagy by 3-MA or siRNA targeted the substantial autophagic gene ATG5, then the occurrence of apoptosis was determined by Annexin-V-FITC/PI assay, the expression of activated caspase-3 and retinoblastoma (Rb) protein was analyzed by western blot.

**Figure 4:**
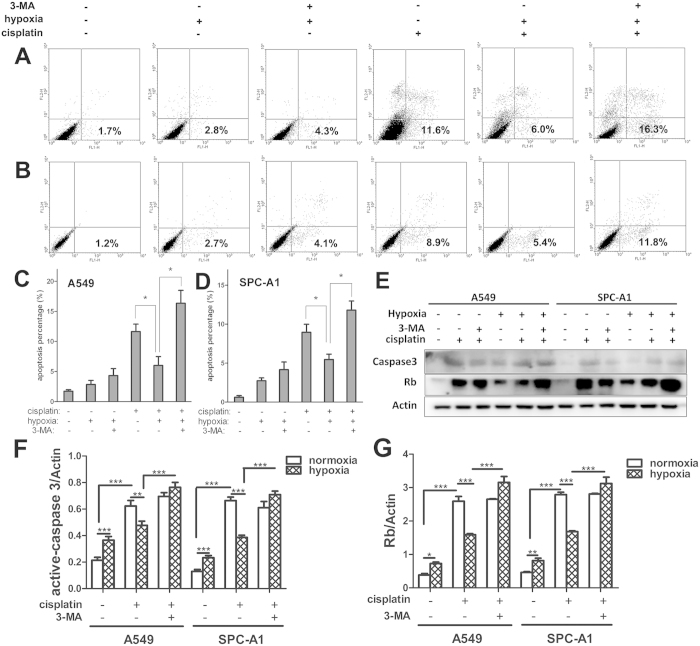
3-MA completely abolished the effect of hypoxia on cisplatin-induced apoptosis. A549 (**A** and **C**) and SPC-A1 (**B** and **D**) cells were treated with or without cisplatin for 12 h under normoxia or hypoxia, followed by Annexin-V and PI staining and FACS analysis. Cells represented early apoptosis (Annexin-V^+^/PI^−^) were calculated (**C** and **D**). (**E**) The expressions of activated-caspase-3 and Rb were detected by western blot. (**F**) The relative expression of activated-caspase-3. (**G**) The relative expression of Rb. Each band was quantified using densitometry. Data were shown as the means ± SD. *p < 0.05, **p < 0.01, ***p < 0.001. All studies were representative of at least three independent experiments. Blot images were cropped for comparison.

First, the percentage of apoptotic cells was analyzed by flow cytometry, as shown in Fig. 4A,B, cisplatin significantly induced apoptosis in A549 and SPC-A1 cells in both normoxic and hypoxic conditions. Compared with that in normoxia, cisplatin-induced apoptosis in A549 and SPC-A1 cells was significantly decreased in hypoxic conditions (Fig. 4A–D). Interestingly, the addition of 3-MA (10 μM) dramatically restored the rate of apoptosis under hypoxia in both A549 and SPC-A1 cells (Fig. 4A–D), comparable to that under normoxia with cisplatin treatment. To further confirm this restoration, siRNA targeted ATG5 was transfected into A549 and SPC-A1 cells. Remarkably, knockdown of ATG5 almost completely abrogated the effect of hypoxia on cisplatin-induced cell apoptosis (Supplementary Fig. 4). The knockdown efficiency of ATG5 siRNA in both A549 and SPC-A1 cells was showed in supplementary Fig. 4F.

Next, we found that cisplatin significantly induced the expression of activated caspase-3 and Rb which both widely observed during apoptosis^29^,^30^ in A549 and SPC-A1 cells under both normoxic and hypoxic conditions. Compared with that in normoxia, cisplatin-induced expression of activated caspase-3 and Rb was significantly decreased in hypoxic conditions (Fig. 4E–G). Interestingly, the addition of 3-MA dramatically restored the expression of activated caspase-3 and Rb under hypoxia in both A549 and SPC-A1 cells (Fig. 4E–G). The similar results were obtained by ATG5 siRNA treatment (Supplementary Fig. 4E). These data implied that autophagy mediates cisplatin resistance of lung cancer cells in hypoxia, inhibition of autophagy restores lung cancer cells sensitivity to cisplatin therapy.

### Hypoxia augmented cisplatin-induced autophagy in lung cancer cells

**Figure 5:**
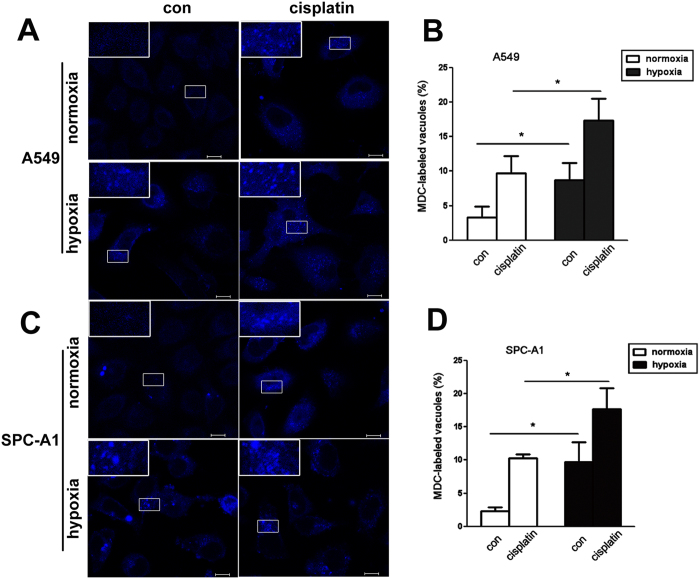
Hypoxia up-regulated the number of autophagolysosome induced by cisplatin. A549 (**A**, upper) and SPC-A1 (**C**, lower) cells were treated with cisplatin for 12 h under normoxia or hypoxia respectively. MDC was loaded into cells and incubated at 37 °C for 15 min, then visualized using confocal laser scanning (400×). The ratio of A549 or SPC-A1 cells containing MDC-labeled vacuoles was calculated and showed in (**B** and **D**). The magnified representative vacuoles were showed upper left. Data were shown as the means ± SD. *p < 0.05.

We then assessed the effect of hypoxia on autophagy in lung cancer cells upon treatment of cisplatin. First, following 12h incubation under normoxia or hypoxia with or without cisplatin treatment, A549 and SPC-A1 cells were stained by MDC, a specific autophagolysosome marker. As shown in Fig. 5A,C, cisplatin significantly induced MDC localization in vacuoles in both A549 and SPC-A1 cells. Compared with that in normoxia, cells under hypoxia exhibited significantly high percentage of cells containing MDC-labeled vacuoles (Fig. 5B,D).

**Figure 6:**
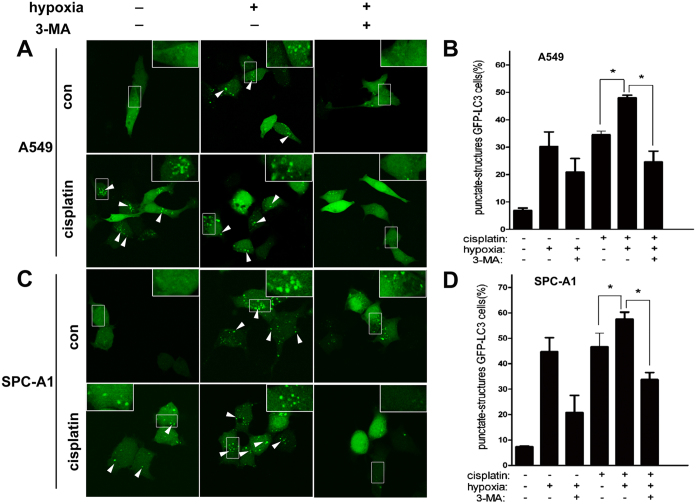
Hypoxia promoted cisplatin-induced LC3 accumulation in lung cancer cells. A549 and SPC-A1 cells were transfected with GFP-tagged LC3. After 24 h transfection, cells were incubated under normoxia or hypoxia in the presence or absence of cisplatin for 12 h. 3-MA was used for inhibiting autophagy. Images were taken by confocal laser scanning microscope. (**A**,**C**) Representative images showed the punctate GFP-LC3 in the cytoplasm. The percentage of cells containing GFP-LC3 puncta in A549 (**B**) and SPC-A1 (**D**) cells was counted and 100 cells were included for each group. The white arrows indicated the GFP-LC3 dots. The results were shown as means ± SD. *p < 0.05.

Sencond, we transfected A549 and SPC-A1 cells with GFP-LC3 plasmids, and analyzed the occurrence of autophagy by monitoring the GFP dots. After transfection, cells were incubated under normoxia or hypoxia in the presence or absence of cisplatin. Then cells were observed by CLSM, and the cells with diffused or punctuate GFP were counted. As shown in Fig. 6A,B, cisplatin increased the GFP-LC3 puncta (as shown by white arrows) under conditions of both normoxia and hypoxia in A549 cells (Fig. 6A,B). However, there were significantly more cells containing GFP-LC3 puncta induced by cisplatin under condition of hypoxia as compared to that in normoxia (Fig. 6A,B). Interestingly, 3-MA significantly reduced the hypoxia or/and cisplatin-induced GFP-LC3 puncta accumulation (Fig. 6A,B). The similar results were showed in SPC-A1 cells (Fig. 6C,D).

**Figure 7:**
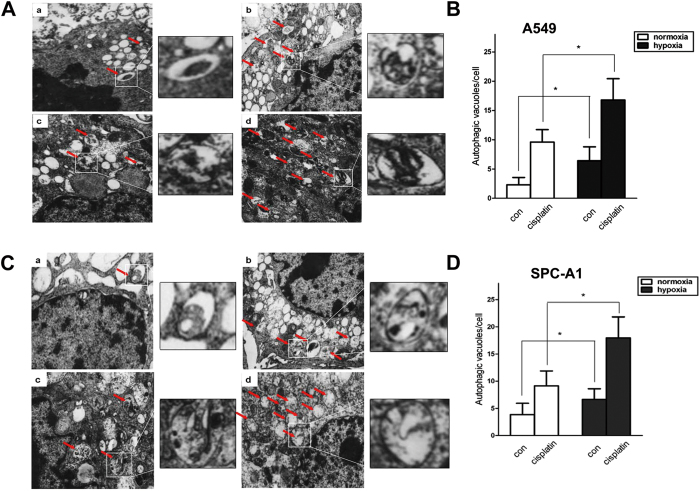
Hypoxia induced more autophagic vacuoles in lung cancer cells treated with cisplatin. A549 (**A** and **B**) and SPC-A1 (**C** and **D**) cells were treated with cisplatin for 12 h under normoxia or hypoxia. Electron micrographs showed the ultrastructure of A549 and SPC-A1 cells. Red arrows indicate the autophagic vacuoles in the cytoplasm. Magnification: 10,000×. The number of autophagic vacuoles per cell was analyzed in A549 (**B**) and SPC-A1 (**D**) cells. 20 cells were investigated per group. Data were shown as the means ± SD. *p < 0.05.

As another independent assay of autophagy, cells were processed for transmission electron microscopy after incubated under normaxia or hypoxia with or without cisplatin treatment. Representative electron micrographs shown in Fig. 7 demonstrated that cisplatin induced more autophagic vacuoles, and the number of cisplatin-induced autophagic vacuoles per cell was significantly increased by hypoxia in both A549 (Fig. 7A,B) and SPC-A1 cells (Fig. 7C,D).

**Figure 8:**
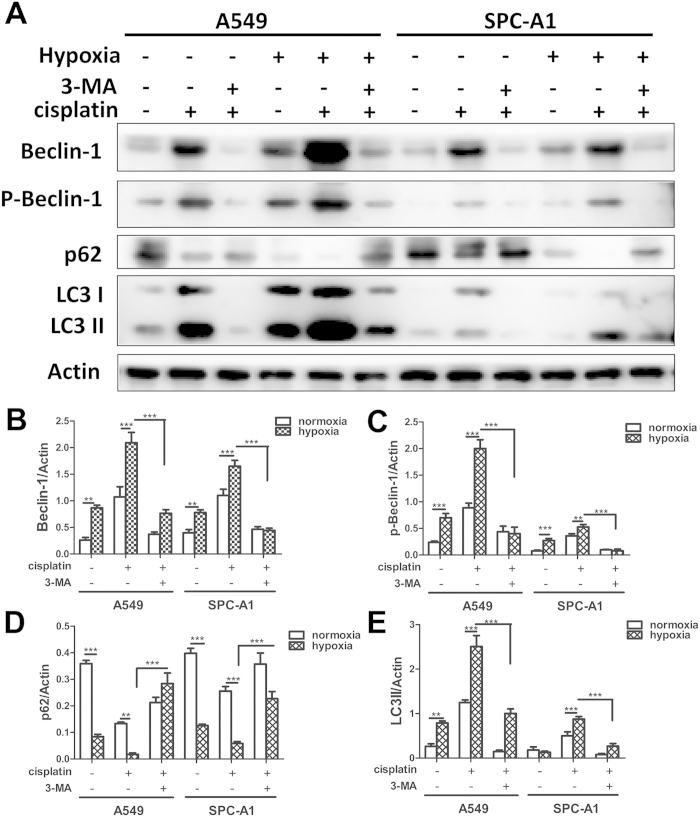
Hypoxia augmented cisplatin-induced Beclin-1, p-Beclin-1, LC3-II abundance and p62 degradation in lung cancer cells. (**A**,**B**) A549 and SPC-A1 cells were incubated under normoxia or hypoxia with or without cisplatin for 12 h. Proteins were extracted and subjected to western blot using Beclin-1, p-Beclin-1, p62 and LC3 antibody (**A**). (**B**–**E**) The relative Beclin-1, p-Beclin-1, p62 and LC3-II expressions were quantified by detecting densitometry. Data were shown as the means ± SD. **p < 0.01, ***p < 0.001. All studies were representative of three independent experiments. Blot images were cropped for comparison.

To further confirm the involvement of autophagy by additionally independent assay, we analyzed the expression of Beclin-1, p-Beclin-1, p62 and LC3-II, which are hallmarks of autophagy by western blot^31^. Compared to that in normoxia, hypoxia markedly increased the levels of Beclin-1, p-Beclin-1 and LC3-II induced by cisplatin in both A549 and SPC-A1 cells (Fig. 8A–C,E), whereas these increases were markedly blocked when simultaneously treated with 3-MA (Fig. 8A–C,E). Additionally, cisplatin significantly decreased the level of p62 in both normoxia and hypoxia conditions, however, there was significant lower level of p62 induced by cisplatin under hypoxia condition as compared to that in normoxia condition (Fig. 8A,D). Cisplatin and 3-MA simultaneously treatment significantly increased the expression of p62 under both normoxic and hypoxic conditions (Fig. 8A,D).

### Hypoxia suppressed cisplatin-induced BNIP3 death pathways

**Figure 9:**
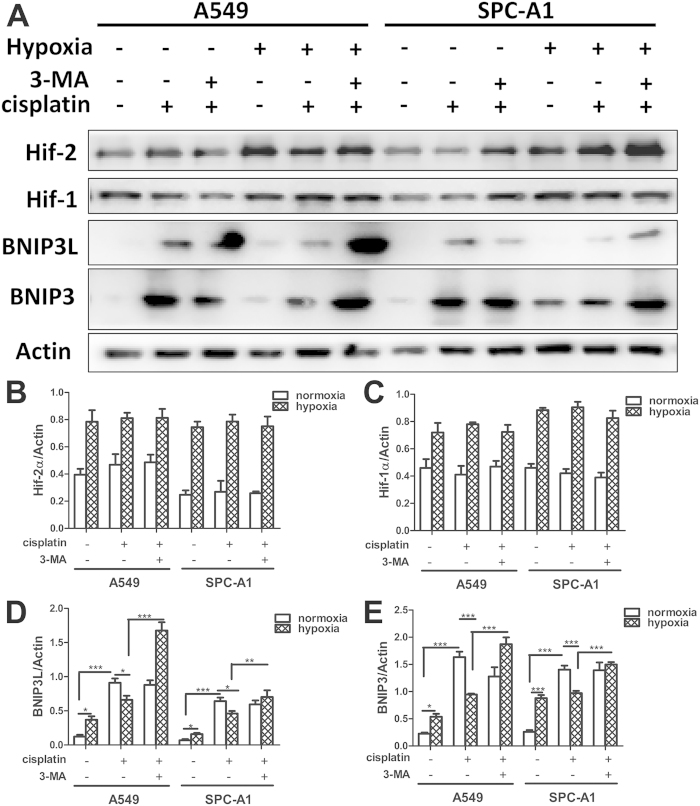
Hypoxia suppressed cisplatin-induced BNIP3 death pathways in lung cancer cells. (**A**,**B**) A549 and SPC-A1 cells were incubated under normoxia or hypoxia for 12 h with or without cisplatin. Proteins were extracted and subjected to western blot using Hif-1α, Hif-2α, BNIP3 and BNIP3L antibody (**A**). (**B**–**E**) The relative expressions of Hif-1α, Hif-2α, BNIP3 and BNIP3L were quantified by detecting densitometry. Data were shown as the means ± SD. *p < 0.05, **p < 0.01, ***p < 0.001. All studies were representative of three independent experiments. Blot images were cropped for comparison.

As for BNIP3 (BCL2/adenovirus E1B 19kDa-interacting protein 3) and its close family member BNIP3L have been identified as apoptotic mediators under hypoxia^32^,^33^, and BNIP3 has been identified as a hypoxia inducible regulator of autophagy^26^. We further studied whether BNIP3 and/or BNIP3L were involved in the hypoxia-induced cisplatin resistance. A549 and SPC-A1 cells were incubated under normoxia or hypoxia with or without cisplatin. It had to be noted that, in these conditions, hypoxia did lead to Hif-1α and Hif-2α activation but cisplatin had no influence on this process (Fig. 9A–C). On the other hand, BNIP3 and BNIP3L abundance was markedly increased by cisplatin or hypoxia, but this abundance was decreased when cells treated with cisplatin under hypoxia (Fig. 9A,D,E). However, the addition of 3-MA completely restored the expression of BNIP3 and BNIP3L (Fig. 9A,D,E).

## Discussion

Hypoxia is a largely studied factor promoting cancer cells chemoresistance. Moreover, autophagy has been highlighted as a cytoprotective mechanism against chemotherapy in these past years^23^. Here, our results demonstrated that robustly augmented-induction of autophagy by hypoxia mediated cisplatin resistance. Targeting autophagy was sufficient to restore lung cancer cells susceptibility to cisplatin.

It was generally acknowledged that, in hypoxic condition, cancer cells undergo a series of genetic and metabolic changes that allow them to be more resistant to chemotherapy^8^,^34^,^35^,^36^. Consistent with these studies, we showed that lung cancer cells incubated with hypoxia showed a lower percentage of cell death compared with that in normoxia when treated with cisplatin. One of the key players for hypoxia-mediated effects is hypoxia-inducible factor (Hif)^27^,^37^. Several studies show that Hif-1α is involved in hypoxia-induced chemoresistance^38^,^39^,^40^,^41^. Our study further revealed that both Hif-1α and Hif-2α were critical for the hypoxia-induced cisplatin resistance.

There are several explanations for hypoxia-induced cisplatin resistance, of which reduced cellular susceptibility to apoptosis may be the most prevailing one^42^. Defective apoptosis underpins cisplatin resistance and is a hallmark of lung cancer^43^. Mitochondria are at the crossroads of apoptotic pathways induced by anticancer agents at several levels. In response to apoptotic stimuli, the outer mitochondrial membrane is permeabilized, causing tanslocation of Bax protein from cytosol to mitochondria and subsequent cytochrome c released from mitochondria into the cytosol where it helps to activate the caspases^31^. However, hypoxia inhibits cell apoptosis by blocking Bax translocation^44^. Consistant with these reports, we demonstrated that cisplatin promoted Bax translocation and Cyto C release, whereas this effect was suppressed by hypoxia. Our result further supported the view that hypoxia decreased lung cancer cells sensitization to cisplatin by affecting their apoptotic potential.

At the same time, it has been reported that tumor resistance to many anticancer agents including cisplatin, tamoxifen can be enhanced through upregulation of autophagy in different tumor cell lines^45^,^46^. Moreover, inhibition of autophagy augments cytotoxicity in combination with several anticancer drugs in preclinical models by triggering apoptosis^47^,^48^. Therefore, the role of autophagy in the hypoxia-induced cisplatin resistance deserves attention. By our analysis, cisplatin induced a significant increase of apoptosis in both A549 and SPC-A1 cells, but the apoptotic rate and the level of active caspase 3 decreased a lot under hypoxic condition. Interestingly, when lung cancer cells were treated by 3-MA or ATG5 siRNA under hypoxia, they became sensitive to cisplatin, similar to the cells under normoxia. Despite present controversies on the exact role of autophagy in the process of tumor generation and progression, either by cell protection or contrarily by inducing cell death^49^,^50^, a majority of studies have been indicated that autophagy is a protective mechanism associated with increased resistance to chemotherapy^2^,^51^. Our results also indicated the protective role of autophagy in the process which hypoxia potentially prevents lung cancer cells from cisplatin-induced apoptosis. Meanwhile, our result demonstrated that cisplatin significantly increased the expression of Rb, while this increase was counteracted by simultaneous hypoxia treatment. However, the addition of 3-MA significantly restored the effect of cisplatin under hypoxia. Many studies have indicated the correlation between Rb and cell cycle control^52^,^53^. Rb has been reported to actively arrest cell cycle progression in G0 or G1 phase when it is unphosphorylated^54^. Therefore, our results indicating that cell survival may be the result of cell cycle arrest under hypoxia and/or cisplatin, while the addition of 3-MA may restore cell cycle progression.

**Figure 10:**
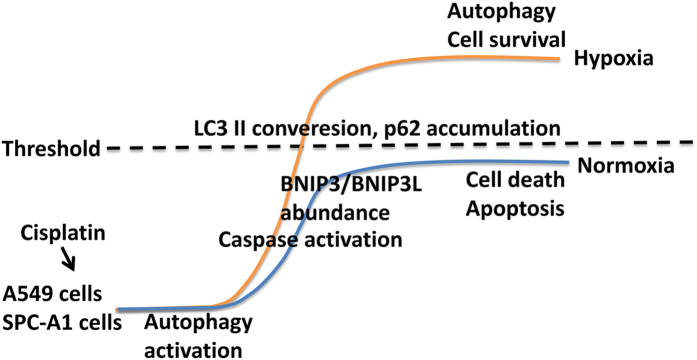
Schematic representation of the lower sensibility of lung cancer cells against cisplatin-induced cell death under hypoxia. Cisplatin induced autophagy activation which was a protective mechanism against cisplatin-induced cell death under both normoxia and hypoxia, and there may be a certain threshold value of autophagy activation. Under normoxia, autophagy activation was unable to excess the threshold to counteract the stress induced by cisplatin, resulting in lower p62 degradation, more BNIP3 and BNIP3L abundance, leading to apoptosis activation and cell death. However, under hypoxia, autophagy induction was augmented that solve the stress, resulting in more p62 degradation, lower BNIP3 and BNIP3L abundance and lower apoptosis activation, allowing the cells to survival.

Some studies have reported that hypoxia can activate autophagy^55^,^56^,^57^, and the hypoxia-induced autophagy may protect cells from apoptosis induced by chemotherapeutic agents^58^. Here, we showed that regardless of O_2_ concentration, cisplatin treatment dramatically increased the accumulation of GFP-LC3 puncta, the formation of autophagic vacuoles, the protein level of Beclin1, p-Beclin1 and LC3-II, as well as the degradation of p62, which are typical features of autophagy. However, note that cisplatin-induced autophagy was markedly augmented by hypoxia. These evidences suggested that there may be a certain threshold value of autophagy activation in lung cancer cells stimulated by cisplatin as shown in Fig. 10. Under normoxia, autophagy activation was unable to excess the threshold, whereas under hypoxia, autophagy was augmented and exceed the threshold. The different regulation of autophagy process by normoxia and hypoxia may be the underlying mechanism for the cisplatin resistance.

As for the molecular mechanism, transcriptional factor Hif-1α and Hif-2α are considered to play foundamental roles under hypoxia^59^,^60^. Hif-1, by regulating the expression of its target downstream BNIP3 and BNIP3L^32^, regulates the activation of apoptosis, autophagy and necrosis under hypoxia^61^. Our study showed that either cisplatin or hypoxia markedly increased BNIP3 and BNIP3L expression. However, their simultaneous treatment reduced the elevation of BNIP3 and BNIP3L induced by cisplatin, and the addition of 3-MA completely restored the elevation. The expression of BNIP3 is reported to be tightly regulated, as its overexpression induces cell death^61^.

Consequently, we hypothesized that autophagy was a protective mechanism after cisplatin incubation under both normoxia and hypoxia, and there may be a certain threshold value of autophagy activation. Under normoxia, autophagy activation was unable to excess the threshold to counteract the stress induced by cisplatin, resulting in lower p62 degradation, more BNIP3 and BNIP3L abundance, leading to apoptosis activation and cell death. However, under hypoxia, autophagy induction was augmented that solved the stress, resulting in more p62 degradation, lower BNIP3 and BNIP3L abundance and lower apoptosis activation, allowing the cells to survival (Fig. 10). Therefore, this would require a better understanding of this signaling pathway involved in autophagic survival and the subsequent cisplatin resistance in future study.

In conclusion, the present study demonstrated that cisplatin resistance in lung cancer cells under hypoxia can be explained by the augmented induction of autophagy, which suppressed BNIP3 death pathway. Further studies on the underlying molecular mechanisms of hypoxia-induced autophagy in chemoresistance will provide novel chemotherapeutic agents for lung cancer therapy.

## Materials and Methods

### Cell culture

The human lung cancer cell lines A549 and SPC-A1 (China Centre for Type Culture Collection, China) were maintained in DMEM (GBICO, Invitrogen,12100-038) containing 10% FBS (GBICO, Invitrogen, 10099-141) at 37 °C in humidified atmosphere of 5% CO_2_. The modular incubation chamber (MIC-101) from Billups-Rothenberg Inc. was used for hypoxic cell culture conditions, the cells were pre-incubated with 1% O_2_ for 1 h before treatment of cisplatin, the detailed protocol and PO_2_ levels of the medium were described in our previous study^62^.

### Cell viability assay

Cell viability was assessed with the MTT assay. A549 and SPC-A1 cells were seeded in 96-well plates at a density of 1 × 10^4^ cells/well and cultured in 1% or 21% O_2_ in medium containing cisplatin (Sigma, P4394) as indicated for 24 h. Next, 10 μl of MTT (5 mg/ml in PBS, Amersco, 0793) was added to each well and incubated for 4 h at 37 °C. Then, the formazan crystals were solubilized with 200 μl DMSO (Sigma, D2650). The absorbance (A) at 570 nm was measured using an automatic multiwell spectrophotometer. The experiment was repeated four times for each group.

### PI staining

A549 and SPC-A1 cells were seeded in 12-well plates containing coverslips at a density of 1 × 10^4^ cells/well and cultured in 1% or 21% O_2_ in medium containing cisplatin (Sigma, P4394) as indicated for 24 h. 1 ug/ml PI (BD Pharmingen™, 556547) was added to each well and incubated for 10 min at 37 °C, then cells were rinsed three times and observed by confocal sanning microscopy.

### Mitochondrial and cytosolic protein fractionation

In order to determine the release of cytochrome C from mitochondria to cytosol and the translocation of Bax from cytosol to mitochondira, the isolation of mitochondria and cytosol was performed using the Cell Mitochondria Isolation Kit (abcam, ab110170). Briefly, 5 × 10^7^ cells were harvested and incubated in 100 μl ice-cold mitochondrial lysis buffer on ice for 10 min. Cell suspension was placed into a glass homogenizer and homogenized for 50 strokes using a tight pestle on ice. The homogenate was then centrifuging at 600 g for 10 min at 4 °C to remove the nuclei and unbroken cells. The supernatant was then collected and centrifuged at 12000 g for 30 min at 4 °C to isolate the cytosol (supernatant) and mitochondria (deposition) fractions. Samples of cytosol and mitochondria were dissolved in lysis buffer and proteins were subjected to western blotting, respectively.

### Immunofluorescence labeling and confocal microscopy

A549 and SPC-A1 cells were seeded in 12-well plates containing coverslips 24 h before indicated treatments. Cells were incubated for 12 h with or without cisplatin under normoxic or hypoxic conditions. After incubation, cells were incubated with 100 nM MitoTacker (Invitrogen, M7513) under growth conditions for 30 min. After staining was completed, the cells were fixed by 10% formaldehyde. Then cells were permeabilized in PBS containing 0.5% Triton X-100 and incubated for 2 h with primary antibody rabbit anti-Bax (abcam,1:200, ab10813) or rabbit anti-Cyto C (abcam, 1:200, ab53056). The second antibody was Alexa Fluor 488®-conjugated anti rabbit IgG antibody (Jackson ImmunoResearch Lab, Inc., 1:1000, 711-545-152). Cells were then washed three times with PBS, the coverslips were mounted and observed with confocal microscopy (60×).

### Apoptosis assay

Apoptosis was detected using an Annexin V-FITC/propidium iodide (PI) staining assay. A549 and SPC-A1 cells were treated with 10 μM and 30 μM cisplatin for 12 h respectively, then cells were harvested. After washed with cold PBS three times, the cells were resuspended in 100 μl 1 × Annexin V-FITC binding buffer followed by staining with Annexin V-FITC/PI (BD Pharmingen™, 556547) at room temperature in darkness for 15 min. Apoptosis cells were then evaluated by gating PI and Annexin V-positive cells on a fluorescence-activated cell-sorting (FACS) flow cytometer (BD Pharmingen™, USA). Results were expressed as the percentage of apoptotic cells at an early stage (PI negative and Annexin V positive). All experiments were performed in triplicate.

### RNA interference

Small interfering RNAs targeting Hif-1α, Hif-2α and ATG5 were purchased from Shanghai GenePharma Corporation. The siRNA sequences used were as follows: Hif-1α siRNA (sense: 5’-GCC ACU UCG AAG UAG UGC UTT-3’ and antisense: 5’- AGC ACU ACU UCG AAG UGG CTT -3’); Hif-2α siRNA (sense:5’- GCG ACA GCU GGA GUA UGA ATT-3’ and antisense: 5’-UUC AUA CUC CAG CUG UCG CTT-3’ ); ATG5 siRNA (sense: 5’- GGU UUG GAC GAA AAC CAA CUU GUU UTT-3’ and antisense: 5’- AAA CAA GUU GGA AUU CGU CCA AAC CTT-3’); Negative control siRNA (sense: 5’-UUC UCC GAA CGU GUC ACG UTT-3’ and antisense: 5’-ACG UGA CAC GUU CGG AGA ATT-3’). Cells were transfected with siRNAs by using Lipofectamine 2000 (Invitrogen, 11668-019) according to the manufacturer’s instructions. The knockdown efficiency was determined by western blot analysis. Three independent transfection experiments were performed.

### MDC staining

A monolayer of cells were cultured for 48 h in 2-well glass-covered chamber slides and then treated with or without cisplatin under normoxic and hypoxic conditions for 12 h. Slides were washed with culture medium without serum. Then, cells were exposed to 50mM MDC (Sigma, 30432), an autofluorescent dye, for 10 min at 37 °C and visualized using confocal laser scanning microscope (Carl Zeiss 710). At least 3 areas per well were analyzed. Two wells were analyzed per treatment and per time. The experiment was repeated for four times. The number of MDC-labeled cells was counted.

### Transient transfection and identification of autophagy

A549 and SPC-A1 cells were transfected with GFP-LC3 plasmid using Lipofectamine 2000 (Invitrogen, 11668-019). After 24 h incubation, A549 and SPC-A1 cells were treated with 10 μM and 30 μM cisplatin for 12 h, respectively. Then, the GFP-LC3 punctate-structures were observed using a confocal laser scanning microscope. The experiment was repeated for four times, more than 100 cells were calculated.

### Transmission electron microscopy (TEM)

The cells were pre-fixed in a solution of 2.5% glutaraldehyde in 0.1M PBS (pH 7.4) for 2 h at room temperature, and post-fixed in 1% osmium tetroxide for 2 h. The samples were dehydrated in increasing concentrations of ethanol (50%, 70%, and 100%) and acetone, and then embedded in Araldite. Fifty to sixty nanometer sections were cut on a LKB-I ultramicrotome and transferred to copper grids, post-stained with uranyl acetate and lead citrate, and examined with a Philips CM-120 transmission electron microscopy.

### Western blot

Cells were homogenized in radioimmunoprecipitation assay (RIPA) buffer (50 mM Tris-HCl, pH 7.4, 0.1% SDS, 1% NP-40, 0.25% sodium deoxycholate, 150 mM NaCl, 1 mM EDTA, 1 mM EGTA, and 1 mM Na3VO4). Prior to homogenization, a protease inhibitor cocktail (Sigma, P2714) was added. Proteins were separated on 12% SDS-PAGE gels and were then transferred to PVDF membranes (Millipore, IPVH00010). Membranes were blocked in 5% bovine serum albumin (Sigma, A4737) and probed with antibodies LC3 (Novus, 1:600, NB100-2220), beclin-1 (abcam, 1:2000, ab62557), p-beclin-1 (cell signal, 1:1000, 12476), p62 (cell signal, 1:1000, 5114), Hif-1α (abcam, 1:1000, ab82832), Hif-2α (Santa Cruz, sc-28706), BNIP3 (abcam, 1:1000, ab10433 ), BNIP3L (abcam, 1:800, ab8399), Bax (abcam, 1:600, ab10813), Cyto C (abcam, 1:800, ab56056), COX IV (abcam, 1:5000, ab33985), anti-activated caspase-3 (abcam, 1:1000, ab2302), Rb (Santa Cruz, 1:1000, sc-74562) and β-actin (abcam, 1:1000, ab3280). Bands were quantified using Image J software.

### Statistical methods

All of the experiments were repeated at least three times. The Data were presented as means ± SD. Statistical analysis was performed using SPSS for Windows, version 11.5. Statistical significance was determined using one-way ANOVA with a post hoc Bonferroni’s test. Significance was set to *p* values < 0.05.

## Additional Information

**How to cite this article**: Wu, H.-M. *et al.* Hypoxia-induced autophagy mediates cisplatin resistance in lung cancer cells. *Sci. Rep.* **5**, 12291; doi: 10.1038/srep12291 (2015).

## Supplementary Material

Supplementary Information
