# Supplementary material for: Hypoxia-induced autophagy mediates cisplatin resistance in lung cancer cells
Source: Sci Rep. 2015 Jul 23;5:12291. doi: 10.1038/srep12291 (PMC4511870; doi:10.1038/srep12291)

Hypoxia-induced autophagy mediates cisplatin resistance in lung cancer cells

Hui-Mei Wu[1], Zi-Feng Jiang[1], Pei-Shan Ding, Li-Jie Shao, Rong-Yu Liu*

Department of Pulmonary, Anhui Geriatric Institute, the First Affiliated Hospital of Anhui Medical University, Jixi Road 218, Hefei 230022, China

[1] The authors contributed equally to this work.

*** Corresponding authors:**

Rong-Yu Liu, Department of Pulmonary, Anhui Geriatric Institute, the First Affiliated Hospital of Anhui Medical University, Jixi Road 218, Hefei 230022, Anhui, P.R. China. Tel: +86-551-62922809; Fax: +86-551-62922342. E-mail address: rongyuliu@163.com

Supplementary Figure. 1: Hypoxia reduced chemosensitivity of lung cancer cells to cisplatin. Lung cancer cell lines A549 (A) and SPC-A1 (B) were incubated under normoxia (21%O_2) and hypoxia (1%O_2) condition with various concentrations of cisplatin for 24 h in the presence or absence of Hif-1α or Hif-2α siRNA. At the end of the treatment, cell viability was assessed by PI staining.

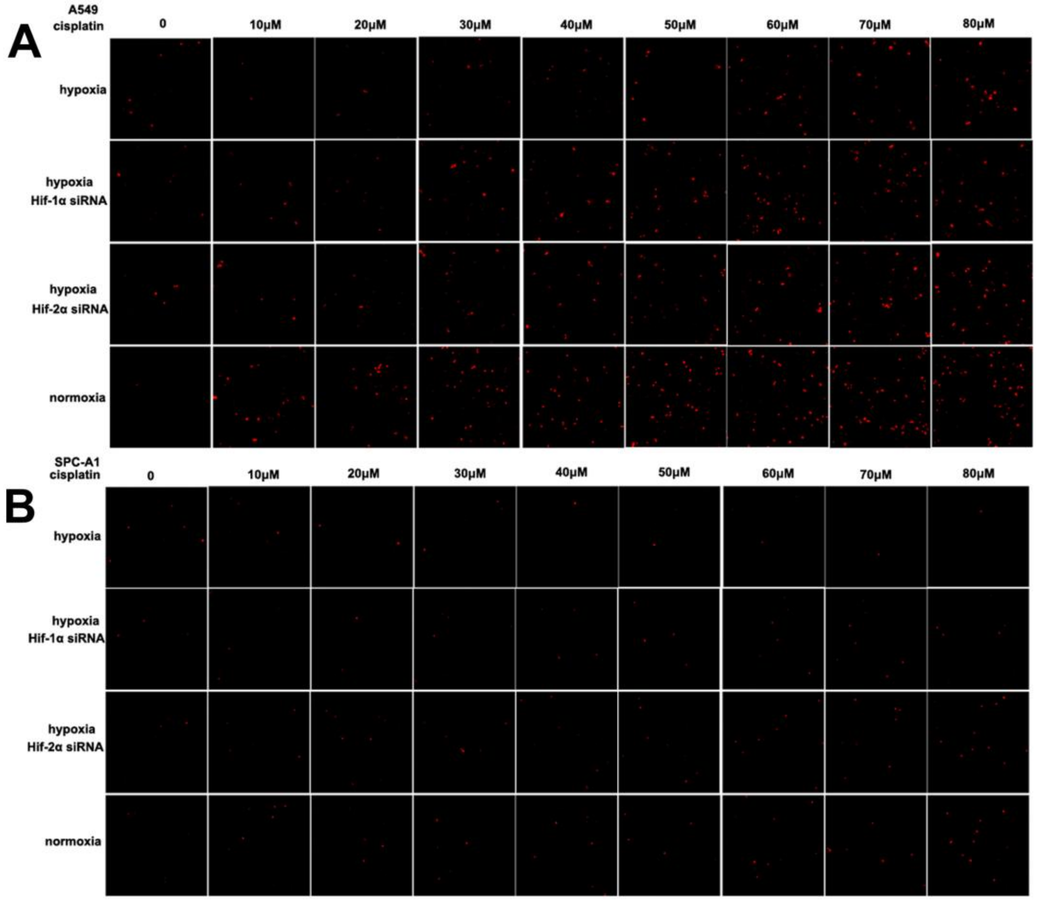

Supplementary Figure. 2: Quantitative analysis of the expression of Hif-1α and Hif-2α at various time points incubated with 1% O_2. (A) Relative expression of Hif-1α in A549 cells. (B) Relative expression of Hif-2α in A549 cells. (C) Relative expression of Hif-1α in SPC-A1 cells. (D) Relative expression of Hif-2α in SPC-A1 cells. The results were shown as means ± SD of four independent experiments. ***p*<0.01，****p*<0.001.

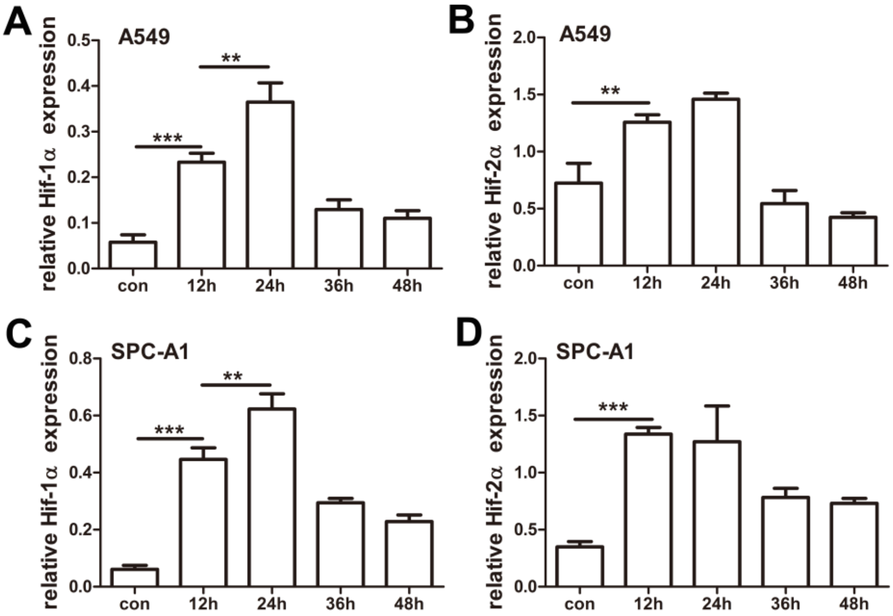

Supplementary Figure. 3: Quantitative analysis of Bax translocation and Cyto C release. A549 and SPC-A1 cells were treated as described, then cells were immunostained by Bax or Cyto C antibody following MitoTracker staining. The percentage of cells containing more than 5 yellow dots (which means colocalization) was counted. (A, B) The percentage of cells containing more than 5 yellow dots in the A549 cells (when cells were labeled by Bax or Cyto C and MitoTracker). (C, D) The percentage of cells containing more than 5 yellow dots in the SPC-A1 cells (when cells were labeled by Bax or Cyto C and MitoTracker). The results were shown as means ± SD of three independent experiments. ***$p<0.001$.

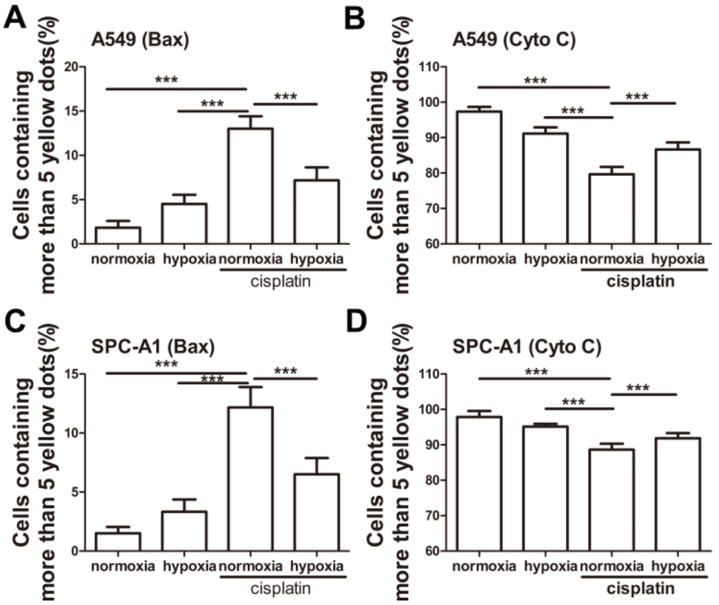

Supplementary Figure. 4: ATG5 siRNA completely abolished the effect of hypoxia on cisplatin-induced apoptosis. A549 (A and B) and SPC-A1 (C and D) cells were treated with or without cisplatin for 12h under normoxia or hypoxia, followed by Annexin-V/PI staining and FACS analysis. Cells represented early apoptosis (Annexin-V$^+$/PI$^-$) were calculated (B and D). (E) The expression of activated-caspase-3 and Rb were detected by western blot. (F) The knockdown efficiency of ATG5 siRNA in the A549 and SPC-A1 cells. Each band was quantified using densitometry. Data were shown as the means ± SD. *p<0.05， **p<0.01.All studies were representative of at least three independent experiments. Blot images were cropped for comparison.

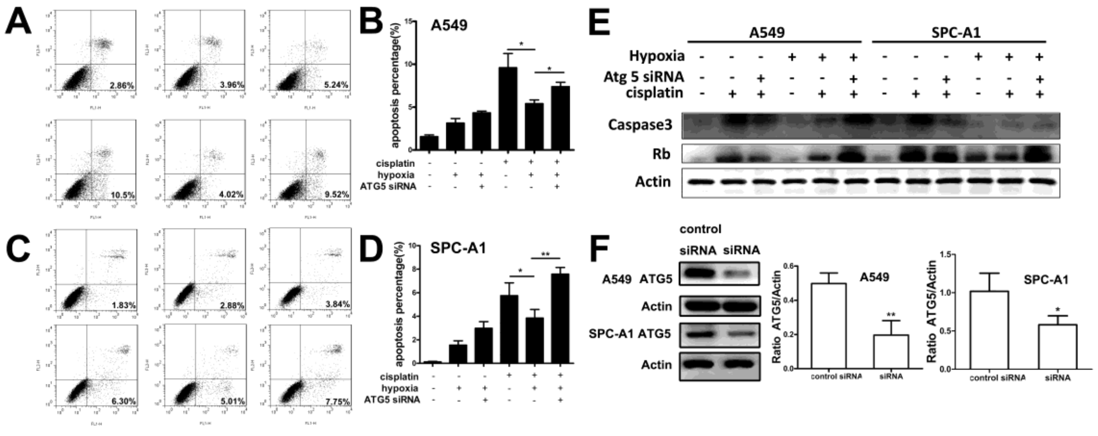

Raw blots for Fig. 1E

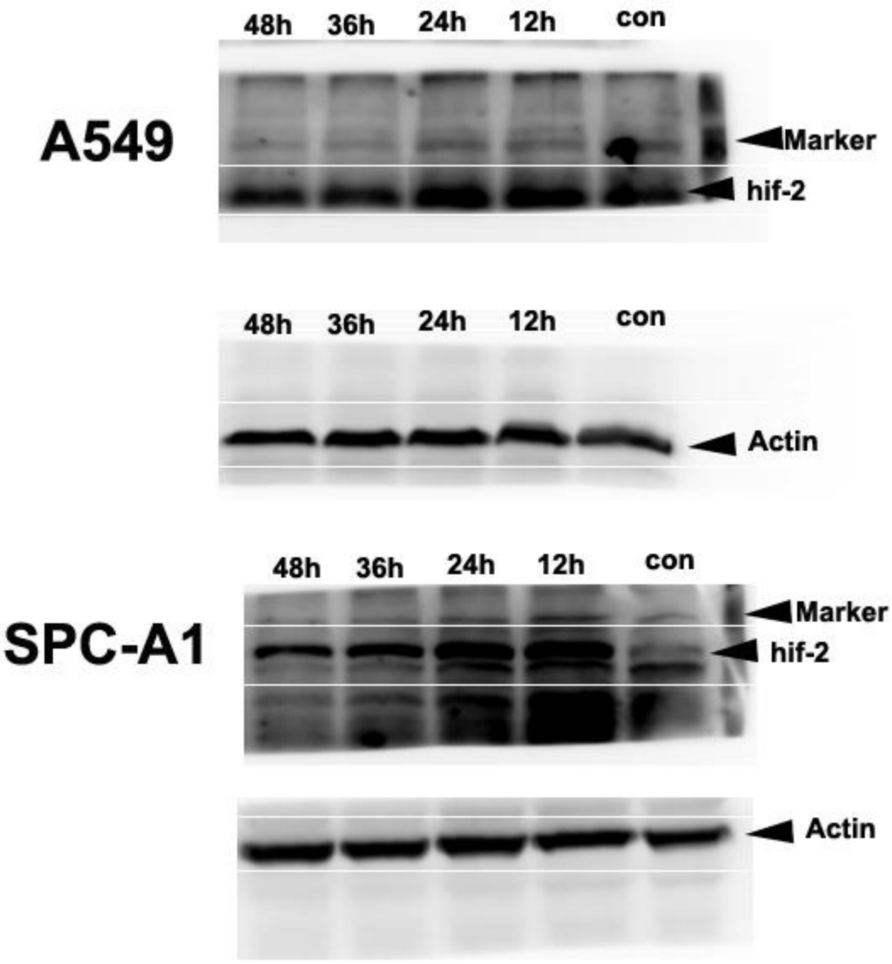

Raw blots for Fig. 2A

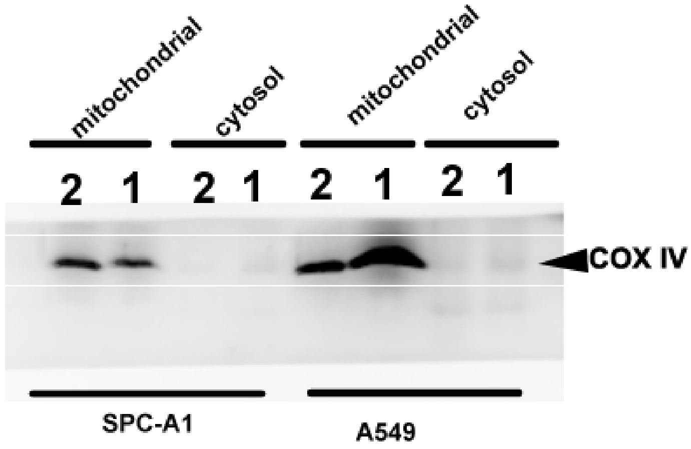

Raw blots for Fig. 4E

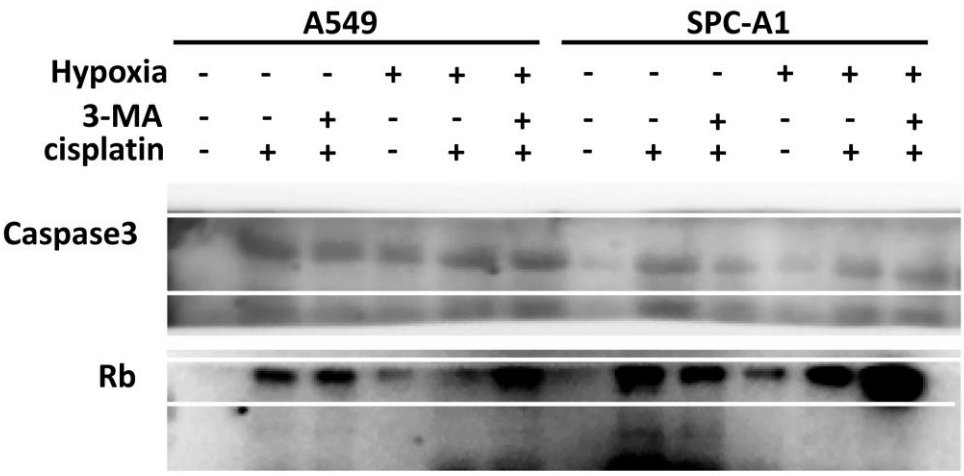

Raw blots for Fig. 8A

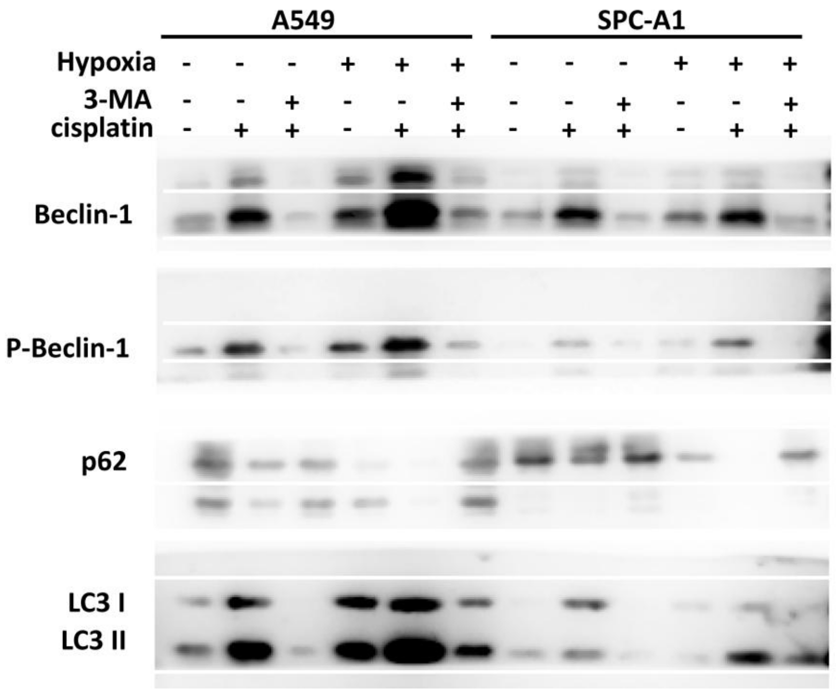

Raw blots for Fig. 9A

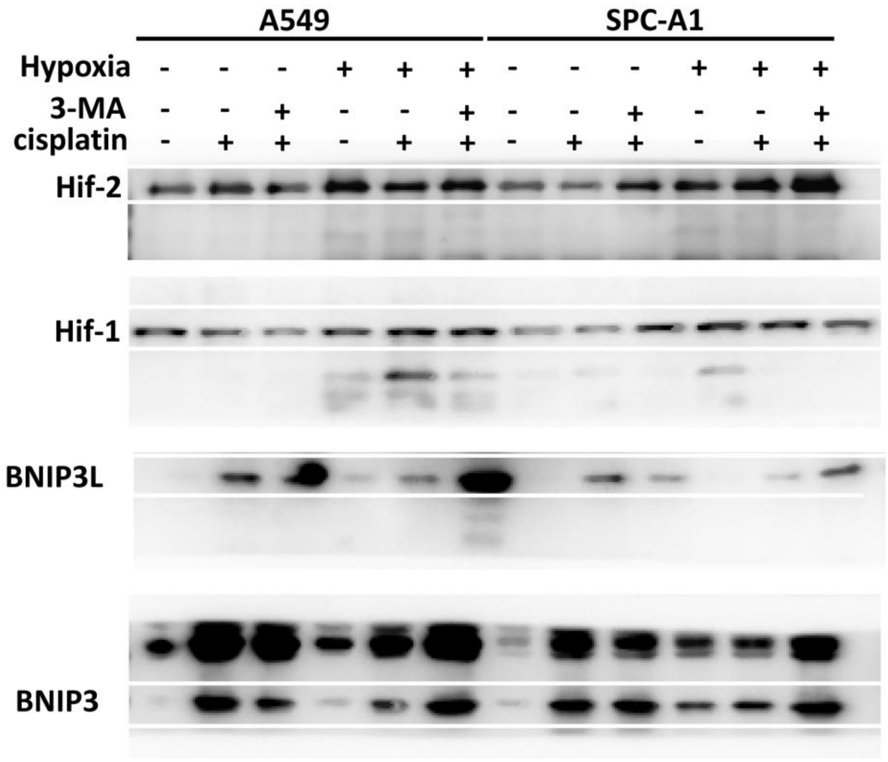

Supplement: Supplementary Information [file srep12291-s1.pdf]
